# Self‐Trapped Excitons in Carbon Quantum Dots with Large NIR‐II Photo‐Thermoelectric Catalysis Induce Pyroptosis for Cancer Therapy

**DOI:** 10.1002/advs.202514249

**Published:** 2025-08-22

**Authors:** Tesen Zhang, Qingcheng Wang, Quansheng Cheng, Han Wang, He Feng, Hui Zhang, Mengbo Lin, Feili Cai, Zheng Fang, Ruo Wang, Gang Chen

**Affiliations:** ^1^ Interdisciplinary Institute of Medical Engineering Fuzhou University Fuzhou 350108 China; ^2^ Institute of Applied Physics and Materials Engineering University of Macau Macau SAR 999078 China; ^3^ Department of General Surgery, Comprehensive Breast Health Center, Ruijin Hospital Shanghai Jiao Tong University School of Medicine Shanghai 200025 China; ^4^ Shengli Clinical Medical College of Fujian Medical University, Department of Breast Surgery, Fujian Provincial Hospital, Fuzhou University Affiliated Provincial Hospital Fuzhou University Fuzhou 350001 China; ^5^ Institute of Molecular Medicine, Renji Hospital, School of Medicine Shanghai Jiao Tong University Shanghai 200127 China; ^6^ Shengli Clinical Medical College of Fujian Medical University, Department of Endocrinology, Fujian Provincial Hospital Fuzhou University Affiliated Provincial Hospital Fuzhou 350001 China

**Keywords:** cancer therapy, carbon dots, NIR‐II photo‐thermoelectric catalysis, pyroptosis, self‐trapped excitons

## Abstract

Pyroptosis, an immunogenic cell death mechanism triggered by Gasdermin family proteins, represents a transformative frontier in tumor immunotherapy. While carbon quantum dots (CQDs) have emerged as pyroptosis‐triggering agents, their efficacy in NIR‐II light‐mediated therapy remains constrained by a low absorption coefficient and uncontrolled charge recombination. Herein, CQDs with high‐density self‐trapped excitons (STEs) exhibiting NIR‐II absorption and an exceptional photothermal conversion efficiency of 51.2% are engineered. By optimizing the migration dynamics of hot carriers, directional charge separation is achieved, which generates cytotoxic hydroxyl radicals and superoxide radicals. The synergistic photo‐thermoelectric catalysis triggers pyroptosis via reactive oxygen species‐caspase 1‐gasdermin D activation, eliciting robust systemic immunity that effectively eliminates the primary tumor and prevents tumor recurrence. This work establishes STE engineering as a universal design principle for advanced nanomaterials while pioneering a NIR‐II‐responsive pyroptosis platform that bridges localized ablation with systemic antitumor immunity, offering a paradigm shift for precision immuno‐oncology.

## Graphical abstract

The self‐trapped excitons in carbon quantum dots can serve as NIR‐II absorbers, which efficiently induce pyroptosis by driving the generation of reactive oxygen species through mild NIR‐II photothermal effect. Combined with local photophysical therapy and the cascade reaction of pyroptosis, it can activate systemic immunity and prevent tumor recurrence. This work provided insights into the development of thermoelectric‐triggered pyroptosis agents.

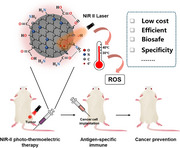

## 1 Introduction

Nanomaterial‐enabled energy conversion therapies (e.g., light, thermal, magnetism) have emerged as a transformative paradigm in tumor physiotherapy, overcoming the physical barriers of conventional treatments to achieve minimally invasive and highly selective tumor ablation.^[^ ^1^ ^]^ This technology demonstrates remarkable advantages, including cost‐effectiveness and broad applicability, positioning it as a pivotal direction for cancer therapy.^[^ ^2^ ^]^ However, there are some critical scientific bottlenecks that persist, i.e., locally confined cytotoxic effects inadequately modulate the tumor immune microenvironment, failing to suppress distant metastasis and recurrence;^[^ ^3^ ^]^ Synergistic mechanisms between physical interventions and systemic immune responses remain insufficiently elucidated, hindering therapeutic paradigm advancement.^[^ ^4^ ^]^ Nanomaterial‐mediated physiotherapy can induce immunogenic cell death (ICD) such as Gasdermin (GSDM)‐activated pyroptosis pathways.^[^ ^5^ ^]^ This process triggers tumor cells' transition from immunologically silent to active states, while releasing damage‐associated molecular patterns (DAMPs) to enhance antigen presentation efficiency and reprogram immunosuppressive tumor microenvironments.^[^ ^6^ ^]^ Such immunomodulatory pyroptosis presents a groundbreaking strategy to reconcile localized ablation with systemic antitumor immunity, potentially resolving long‐standing challenges in metastatic control and therapeutic resistance.

Pyroptosis, a pro‐inflammatory programmed cell death first described by Cookson et al. in 2001,^[^ ^7^ ^]^ manifests through distinct morphological features including membrane perforation, osmotic swelling, and cytoplasmic content release.^[^ ^8^ ^]^ This process is molecularly orchestrated by caspase‐mediated proteolytic cleavage of GSDM proteins, generating N‐terminal fragments that oligomerize to form plasma membrane pores.^[^ ^9^ ^]^ Subsequent efflux of proinflammatory cytokines (e.g., IL‐1β and IL‐18) potentiates tumor microenvironment remodeling and systemic antitumor immunity, positioning pyroptosis induction as a strategic paradigm in cancer immunotherapy.^[^ ^8^, ^10^ ^]^ While current approaches employ pharmacological agents,^[^ ^11^ ^]^ bacterial components,^[^ ^12^ ^]^ or reactive oxygen species (ROS)‐generating nanomaterials activated by external fields,^[^ ^13^ ^]^ critical limitations persist. Transition metal‐based nanosystems and perovskite materials face translational barriers due to inherent biosafety concerns,^[^ ^13b,c^ ^]^ whereas organic photosensitizers suffer from low dispersion and photostability.^[^ ^13^, ^14^ ^]^ These constraints underscore the urgent need for developing pyroptosis‐triggering agents (PTAs) that combine precision activation, biosafety, and microenvironmental responsiveness.

The 2023 Nobel Prize in Chemistry‐awarded discovery of quantum dots (QDs) has revolutionized biomedical research.^[^ ^15^ ^]^ Among the various types of QDs, carbon QDs (CQDs) emerged as frontrunners in nanomedicine due to their excellent biocompatibility and multifunctional optoelectronic properties.^[^ ^16^ ^]^ Leveraging tailored photoluminescence, photothermal, and photocatalytic properties, CQDs have demonstrated exceptional prowess in bioimaging and phototherapy.^[^ ^17^ ^]^ However, their potential in thermoelectric catalysis remains underexplored, particularly regarding pyroptosis induction due to their hot charge carrier mobility. CQDs should belong to the time‐reversal fermionic system, where the hot electron mobility surpasses that of hot holes, thereby prolonging the recombination time of hot electron flow and hole flow.^[^ ^18^ ^]^ Efficient charge separation can result in the generation of ROS such as hydroxyl radicals, singlet oxygen, and superoxide radicals, which can trigger pyroptosis.^[^ ^19^ ^]^ Our group has successfully pioneered pyroptosis vaccination through white light‐induced pyroptosis using photocatalytic CQDs.^[^ ^20^ ^]^ However, the limited penetration ability of white light often hinders the effective catalytic‐induced pyroptosis within biological organisms. Therefore, by constructing the NIR‐II absorption band for CQDs and harnessing their photo‐thermoelectric catalysis to achieve pyroptosis efficiently in vivo, significant implications for clinical medical applications could be realized.

In this study, we developed self‐trapped excitons (STEs) of CQDs with NIR‐II absorption and an exceptional photothermal conversion efficiency of 51.2%. Through precisely controlled thermal gradients, we orchestrated directional hot electron flow migration with exceptional carrier mobility, achieving spatiotemporal control, sustained charge separation that drives thermocatalytic hydroxyl radical and superoxide radical generation, which overcome the constraints of conventional band requirements. This radical flux triggers spatially controlled pyroptosis via ROS/caspase‐1/GSDMD axis activation, as demonstrated through the therapy efficiency of tumor‐bearing mice. The pyroptosis‐driven immunogenic cascade elicited systemic antitumor immunity and complete suppression of recurrence. This immune response exhibits specificity, exclusively targeting the same type of tumor being treated, thereby providing a rationale for the development of novel personalized vaccines. Our findings establish STE modulation as a universal design principle for thermoelectric nanomaterials, while pioneering a noninvasive immunotherapeutic modality with NIR‐II light activation.

## 2 Result

### 2.1 The Formation of STEs in CQDs

**Figure 1:**
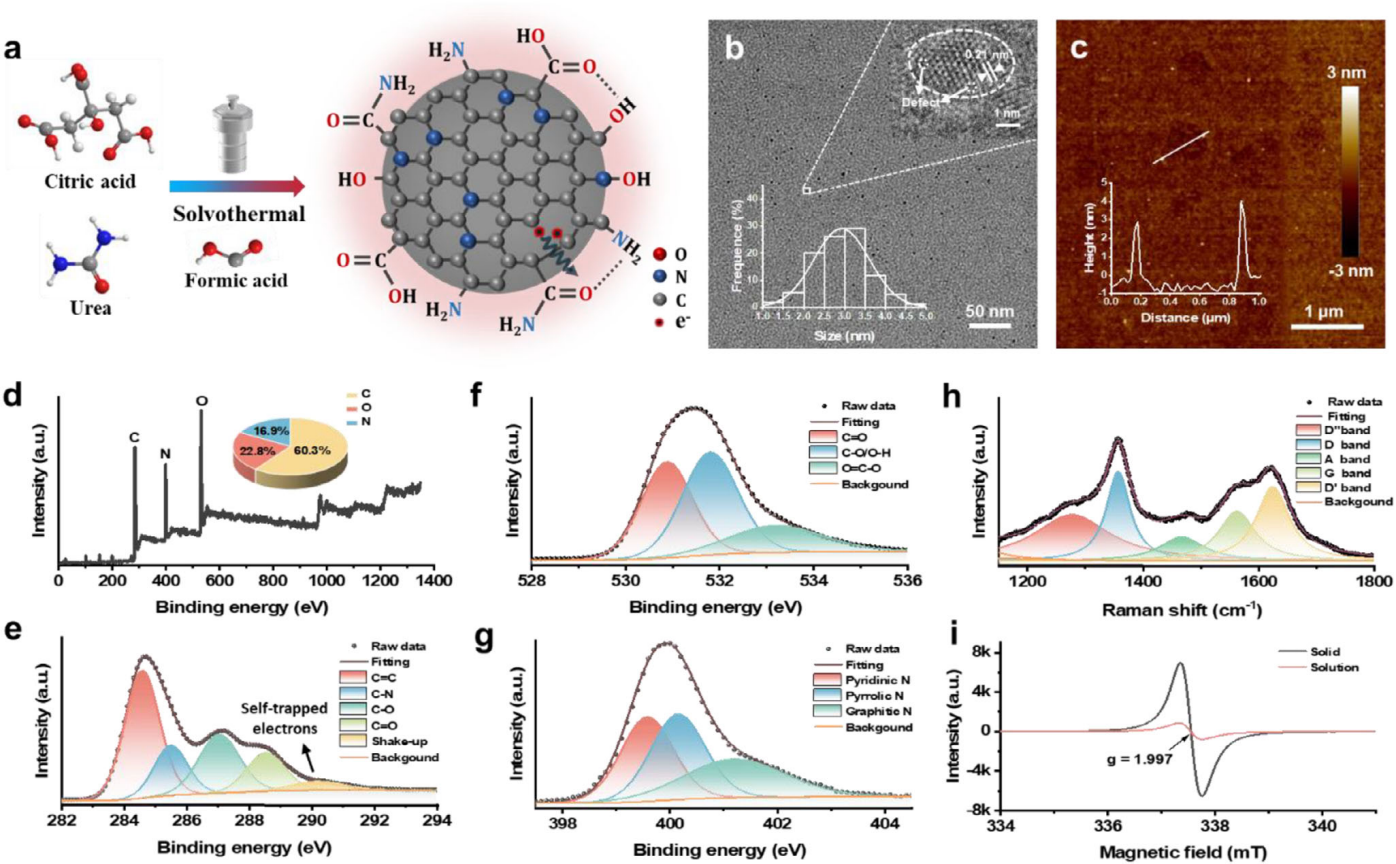
Morphological and structural characterization. a) Schematic illustration of the synthesis of CQDs. b) TEM and HRTEM (top right corner inset) images of CQDs. Bottom left corner inset: particle size distribution chart. c) AFM images of CQDs. Inset: height profile along the line. d–g) XPS survey spectra of CQDs. High‐resolution e) C 1s, f) O 1s, and g) N 1s XPS spectra of CQDs.; h) Roman spectra of CQDs. i) EPR spectra of CQDs in solid state and aqueous solution. a.u., arbitrary units.

CQDs were prepared from 2 g of citric acid and 4 g of urea in formic acid solution by a solvothermal method based on free radical polymerization (**Figure** **1**a). Notably, compared to established methods employing extended π‐conjugated precursors, the use of small molecules as raw materials for one‐step synthesis of NIR‐II‐absorbing CQDs presents a more challenging approach. Citric acid and urea undergo dehydration‐condensation reactions in formic acid under elevated temperatures, forming CQDs with extended conjugation domains and strong electron‐withdrawing ligand environments. Through systematic optimization of precursor ratios and solvothermal conditions, we confirmed that the optimal ratio of citric acid to urea is 1:2, and the solvent‐thermal temperature exceeding 180 °C maximizes the generation of STE density and corresponding NIR‐II absorption. We characterized the morphologies of CQDs using transmission electron microscopy (TEM) and atomic force microscopy (AFM). As shown in Figure 1b, the diameters of CQDs are 3.33 ± 0.54 nm. Similar well‐resolved lattice fringes with an interlayer spacing of 0.21 nm were observed in the high‐resolution TEM (HRTEM) images, indicating graphite‐like core structures in the CQDs. Upon careful observation, more lattice defects (mismatching and vacancies) were noticed in the cores of the CQDs. These defects provided the foundation for the formation of STEs. AFM imaging revealed that the height of CQDs was ≈3 nm (Figure 1c), corresponding to a spherical morphology. We performed X‐ray Photoelectron Spectroscopy (XPS) to confirm the chemical structure. The XPS spectra revealed the presence of C (285.3 eV), N (400.2 eV), and O (531.5 eV) elements (Figure 1d; Table S1, Supporting Information). High‐resolution C 1s, O 1s, and N 1s XPS spectra respectively confirmed the existence of C═C (284.5 eV), C═N (285.2 eV), C─O (286.8 eV), C═O (288.5 eV) and shake‐up peak (290.6 eV); C═O (531 eV), C─O/O─H (532 eV), and O═C─O (533.7 eV); and Pyridinic N (399.4 eV), Pyrrolic N (400.1 eV), and amino N (401.2 eV) in the CQDs (Figure 1e–g). The shake‐up peak in the high‐resolution C1s spectra may arise from the excitation of STEs to higher energy levels when inner‐shell electrons are excited and emitted by X‐rays, resulting in partial energy loss of the photoelectrons. This provides compelling evidence for the presence of STEs. The lattice structure of CQDs was further characterized by Raman spectra. We observed two peaks at 1360 and 1580 cm^−1^ corresponding to the D band and G band, while a broad peak ≈2700 cm^−1^ was attributed to the 2D band (Figure S1, Supporting Information). The high intensity ratio of the D and G bands (I_D_ / I_G_ = 1.17) and the broadening of the 2D peak indicated the disorder of the sp^2^‐domains and the introduction of local lattice distortion. Figure 1h showed five deconvoluted peaks at 1277, 1357, 1468, 1563, and 1625 cm^−1^ marked as D*, D, A, G, and D’ bands are attributed to disordered graphitic lattice, defect or edge‐induced two‐phonon scattering, amorphous carbon in sp^3^ hybridization of carbon polymorphs, in‐plane stretching modes of sp^2^‐domains, and defect‐related phonon modes of sp^2^‐domains, respectively. The appearance of D*, A, and D’ bands indicated that the complex internal defects and highly oxidized edges of CQDs break the time reversal symmetry, which provided main evidence for the formation of STEs. The properties of unpaired electrons were characterized by electron paramagnetic resonance (EPR) spectra (Figure 1i). It has been reported that STEs are formed from free radical’ sites.^[^ ^21^ ^]^ The EPR signal at g = 1.997 can be identified as free radicals or spin defects, which is considered a prerequisite for the formation of STEs in CQDs. Based on the above results, we have synthesized CQDs with high‐density STEs, providing structural support for the subsequent application of photo‐thermoelectric catalysis.

### 2.2 Excited State Dynamics and Thermoelectric Catalytic Mechanism of STEs

**Figure 2:**
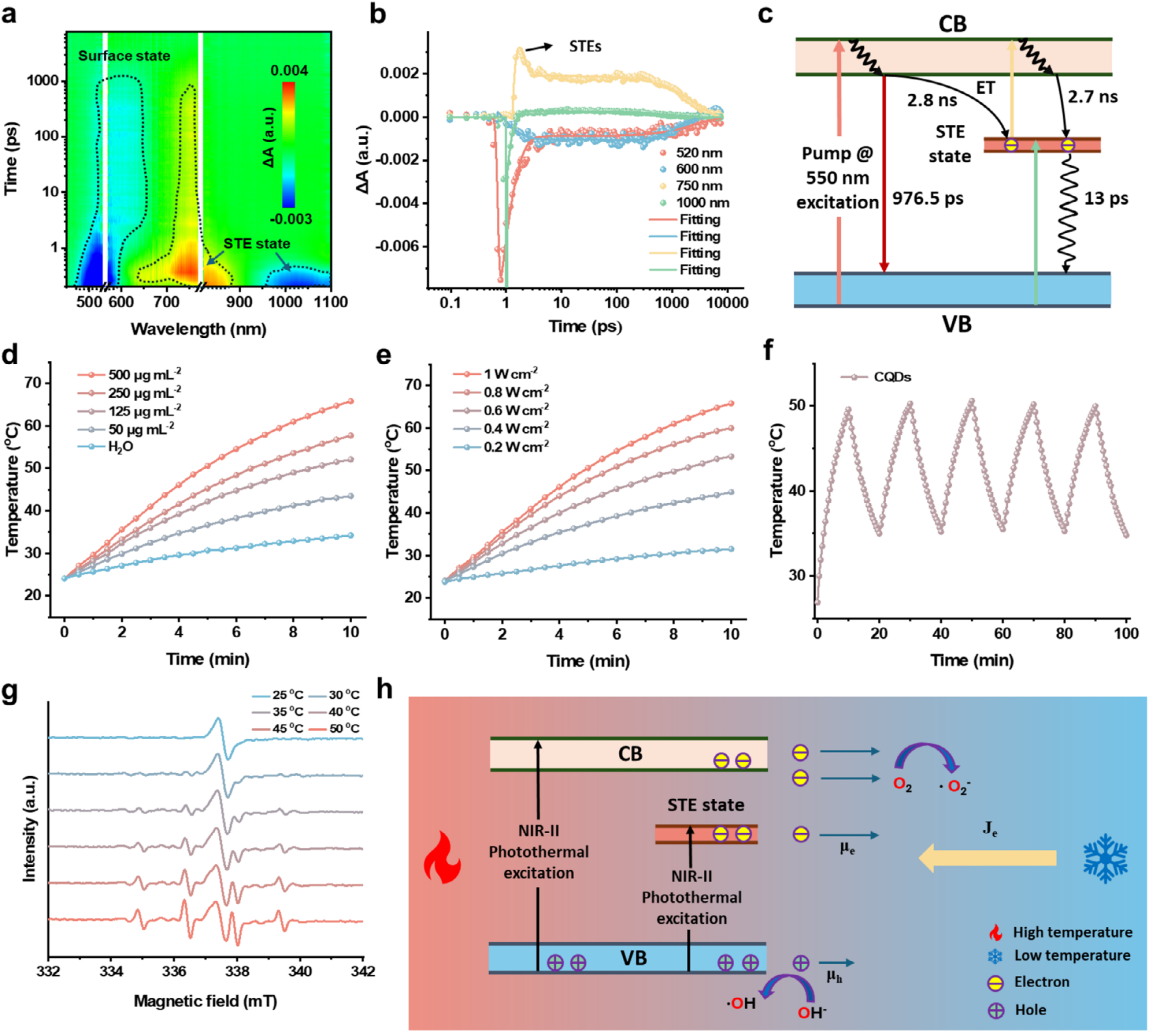
Excited state dynamics and thermoelectric catalytic mechanism of STEs. a) 2D pseudo‐color maps of the TA spectra of CQDs in aqueous solution with a pump wavelength of 550 nm (1 kHz, 100 fs). b) Bleach signal kinetics of CQDs collected at 520, 600, 750, and 1000 nm for λ_pump_ = 550 nm in aqueous solution. Dots are experimental points, and solid lines are fittings. c) Energy level diagrams of the CQDs under λ_pump_ = 550 nm excitation. d) Temperature evolutions of the CQD aqueous solutions at various concentrations and pure water under 1064‐nm laser irradiation at 1 W cm^−2^. e) Temperature evolutions of the CQD aqueous solution at 500 µg mL^−1^ under 1064‐nm laser irradiation at various power densities. f) Temperature curves of the CQD aqueous solution at 500 µg mL^−1^ under five cycles of photothermal heating under 1064‐nm laser irradiation (0.6 W cm^−2^). g) EPR spectra of CQDs in aqueous solution after irradiation by NIR‐II light for different temperatures. h) Energy level diagrams and the thermoelectric catalysis processes under NIR‐II photothermal excitation. µ_e_, electron mobility. µ_h_, hole mobility. J_e_, Current density.

We conducted transient absorption (TA) measurements to investigate the photophysical details of the CQDs in aqueous solution. The CQDs exhibited a ground‐state bleaching (GSB) signal from 950 to 1100 nm and a photoinduced absorption (PIA) signal in the range of 700–880 nm, corresponding to the STE state. While the GSB signals observed in the range of 450–650 nm were attributed to core or surface state (**Figure** **2**a). By monitoring TA kinetic traces probed at 520, 600, 750, and 1000 nm, we can speculate that the energy of the core or surface state is transferred to the STE state (Figure 2b). The rapid rise in the PIA curve at 750 nm is attributed to lattice distortion and electron‐phonon coupling, reflecting the formation of STEs. The subsequent slow decay process likely originates from the localization of excitons in the lattice, which hinders rapid recombination. This is ascribed to the multi‐phonon involvement resulting from the strong coupling between excitons and the lattice. The decay process of the STE state at 1000 nm revealed that these features returned to the ground state in ∼13 ps, which was attributed to the rapid interaction of electrons and phonons (Figure 2c). Therefore, we speculate that the STE state will have excellent photothermal performance under NIR‐II light irradiation.

Then we evaluated the photothermal performance of a CQD aqueous solution. After 10 min of NIR‐II irradiation, the temperature of the CQD aqueous solution (500 µg mL^−1^) rapidly increased by 41.7 °C from room temperature, while the temperatures of pure water only increased by 10.1 °C (Figure S2, Supporting Information). The calculated photothermal conversion efficiency (PCE) of the CQDs reached a high value of 51.2% though a photothermal cooling curve (Figure S3, Supporting Information). Both increasing the concentration of CQD solution and enhancing the power of NIR‐II laser irradiation can notably improve the photothermal effect (Figure 2d,e). Furthermore, the CQDs demonstrated excellent photothermal stability in aqueous solution (Figure 2f). After five cycles of 1064‐nm laser heating and natural cooling, no substantial degradation of the photothermal performance occurred, highlighting the robustness of the CQDs as a promising photothermal agent for cancer therapy.

Finally, we investigated the capacity of NIR‐II photo‐thermoelectric catalysis of CQDs and proposed a possible mechanism. By investigating the EPR spectra with different temperatures under NIR‐II light irradiation, we found that CQDs can generate a greater amount of hydroxyl radicals and superoxide radicals as the temperature increases (Figure 2g; Figure S4, Supporting Information). However, under low‐temperature NIR‐II irradiation, the production of hydroxyl radicals and superoxide radicals is almost completely suppressed. Nevertheless, CQDs retain the ability to produce trace hydroxyl radicals and superoxide radicals at ambient temperature, demonstrating nanozyme‐like characteristics. From the former, the decay process of NIR‐II region, we know that the relaxation time is ≈13 ps. Such rapid optical relaxation times generally make effective charge separation challenging. Thus, we attributed this to the difference in the migration speeds of the hot carriers. Based on these results, we proposed a possible mechanism (Figure 2h). In the CQD system, temperature gradients can alter the Fermi‐Dirac distribution, resulting in the equal generation of electrons and holes. When electrons diffuse from the hot end to the cold end, their migration is more pronounced, leading to more efficient charge separation. Additionally, the mechanistic schematic diagram illustrates that the excited‐state electrons tend to interact with the lattice environment and undergo rapid vibrational relaxation in water. The holes are more prone to reacting with water to generate hydroxyl radicals, and the electrons tend to react with oxygen to generate superoxide radicals. This also indicates that CQDs can function as type I photosensitizers, unaffected by tumor hypoxia and the acidic microenvironment. Consequently, this process afforded CQDs potent catalytic capabilities, laying the foundation for subsequent thermoelectric catalytic therapy.

### 2.3 Fluorescence and Biosafety Assessment

We evaluated the optical properties of CQDs in solutions to assess their biosafety. In aqueous solution, CQDs exhibited a main absorption peak at 560 nm and a broad absorption band at 800–1300 nm. In DMSO solution, due to the influence of zero‐field splitting and solvent effects, CQDs have absorption peaks at 570, 600, 700, 820, 910, and 1030 nm (Figure S5a, Supporting Information). The emission center of CQDs was mainly concentrated in the red‐light region, with an emission peak at 640 nm in DMSO, a fluorescence lifetime of 5.39 ns, and a photoluminescence quantum yield (PLQY) of 68.5%. In aqueous solution, the emission center was at 610 nm, with a fluorescence lifetime of 2.05 ns and a PLQY of 19.2%, which can meet the requirements for bioimaging (Figure S5b–f and Table S2, Supporting Information).

**Figure 3:**
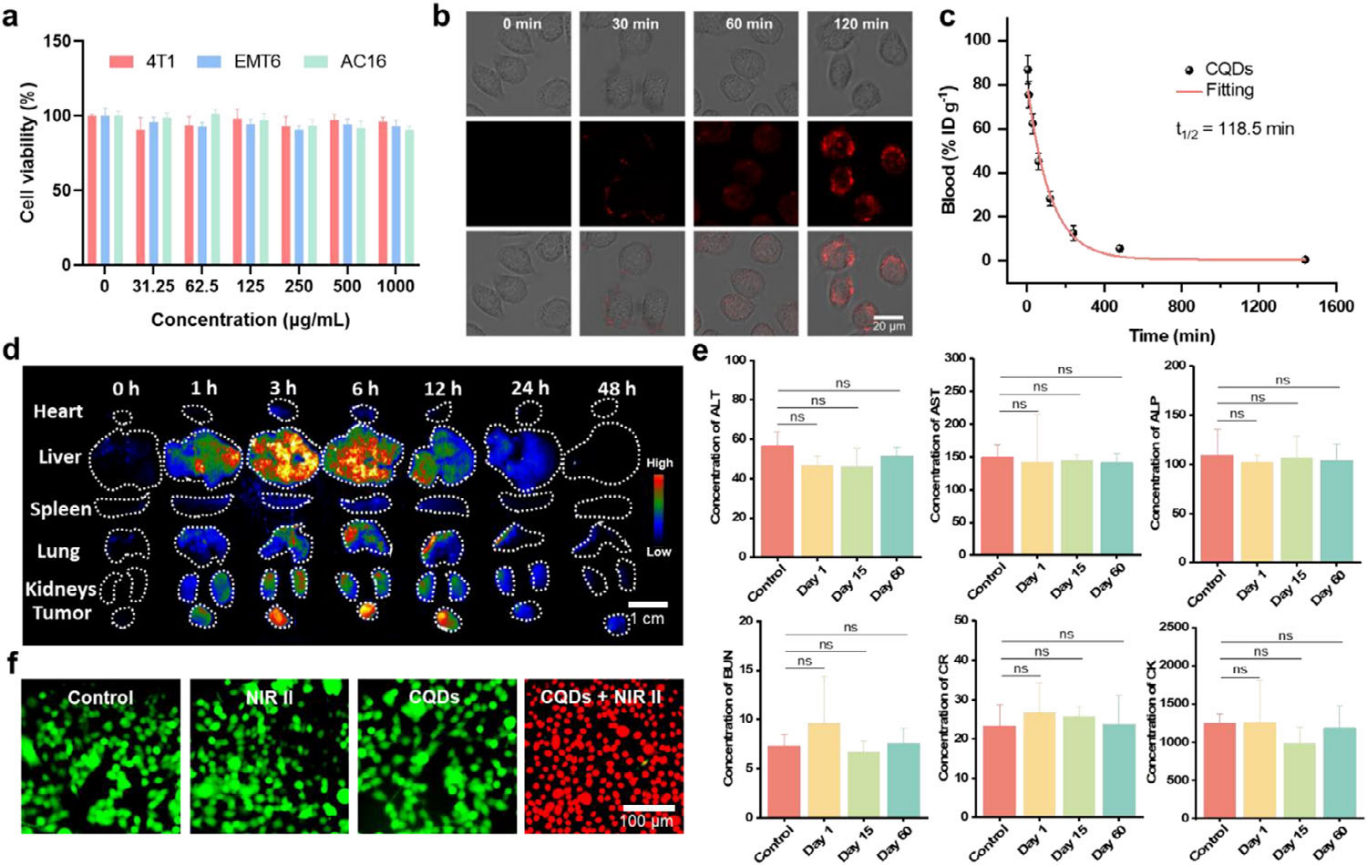
Fluorescence and biosafety assessment. a) Cell viabilities of different cell groups incubated with CQDs of different concentrations. b) Cellular uptake of the CQDs by 4T1 cells after 0, 30, 60, and 120 min. of incubation in a Mchery‐1 channel. c) Blood circulation (%ID g^−1^) of CQD administered mice as a function of time. d) PL imaging of the major mouse organs conducted at different time points before and after intravenous injection of 100 µL of CQD aqueous solution (1000 µg mL^−1^). (Ex, 589 nm laser; Em, 650 nm longpass [LP] optical filter). e) Biochemistry tests of the mice in CQDs group on days 0, 1, 15, and 60 days. ALT (U/L): Alanine transaminase; AST (U/L): Aspartate aminotransferase; ALP (U/L): Alkaline phosphatase; BUN (mmol/L): Blood urea nitrogen; CR (mmol L^−1^): Creatinine; CK (mmol L^−1^): Creatine kinase test, *n*  = 3, two‐way ANOVA multiple comparison test, the absence of a *p*‐value indicates no statistical difference between the groups (*p* > 0.05)). f) Calcein AM and PI assay of EMT6 cells treated with CQDs (500 µg mL^−1^) after a 1064‐nm laser irradiation (0.8 W cm^−2^) for 10 min.

Then the biosafety and biocompatibility of the CQDs were evaluated in vitro and in vivo. The cytotoxicity of the CQDs at various concentrations was assessed in 4T1, EMT6, and AC16 cells using a CCK‐8 assay. After incubating for 48 h, the CQDs did not reduce cell viability at concentrations up to 1000 µg mL^−1^, highlighting their low cytotoxicity (**Figure** **3**a). The cell fluorescence imaging capability of the CQDs was also assessed in EMT6 cells. Following incubation with the CQDs (500 µg mL^−1^) for ≈2 h, gradually enhanced red emission was observed in the EMT6 cells, indicating successful cellular uptake of the CQDs (Figure 3b). Then we evaluated the biodistribution of the CQDs in mice in vivo by red photoluminescence (PL) imaging (Figure 3d; Figure S6, Supporting Information). Mice were intravenously injected with an aqueous solution of CQDs (100 µL, 1000 µg mL^−1^) via the tail vein. Over a period of 0 to 48 h postinjection, major organs, including the heart, lung, liver, spleen, and kidney, were excised for ex vivo red PL imaging to quantify the PL intensity. Before injection, the major organs did not exhibit red PL under 589 nm excitation. However, following the injection of the CQDs, red PL signals were detected in the Tumors, liver, lung, and kidneys. This signal gradually decreased and completely disappeared after 48 h, indicating that the CQDs primarily accumulated in the liver and kidney shortly after injection. The observed passive targeting differences between the lungs and the tumor may result from CQD size enlargement (≈50–120 nm), caused by aggregation or protein corona formation in serum (Figure S7, Supporting Information). Crucially, the metabolism or clearance of larger CQD aggregates in the lungs does not impede the accumulation observed at the tumor site. This transient retention within the tumor, facilitated by the enhanced permeability and retention effect, establishes the essential condition enabling the subsequent therapeutic efficacy demonstrated via tail vein administration in mice. Then we investigated the pharmacokinetics of CQDs in living mice after intravenous injection, and venous blood samples at various time points were collected to measure the CQD contents. The blood half‐life time of 118.5 min indicated the rapid metabolism of CQDs (Figure 3c). These findings suggest that the CQDs were rapidly excreted through the kidney after intravenous administration, demonstrating their excellent biocompatibility and minimal or no biotoxicity. Moreover, CQDs can accumulate in tumors through the enhanced permeability and retention effect, reaching peak levels within 6 hours and exhibiting gradual excretion, thereby providing a solid foundation for NIR‐II cancer therapy. Besides, blood samples were collected from mice at 1, 15, and 60 days using 1000 µg mL^−1^ CQD aqueous solution before NIR‐II cancer therapy. Biochemistry tests were conducted on these samples, with untreated CQD mice serving as controls. The results of these tests are presented in Figure 3e. It is noteworthy that all blood parameters (ALT, AST, ALP, BUN, CR, CK, and LDH) showed no statistically significant differences compared to the control group. Taken together, these findings indicate that there was minimal to no significant inflammation or infection in mice treated with the synthesized CQDs as photosensitizers. The results strongly support the safety and potential clinical applicability of CQDs.

The cytotoxicity of the CQDs under NIR‐II irradiation was assessed via a live/dead cell viability staining assay. Untreated EMT6 cells and nonirradiated cells incubated with CQDs at 1000 µg mL^−1^ showed green fluorescence, indicating cell viability. However, when subjected to irradiation with a 1064 nm laser at 0.8 W cm^−2^, the majority of EMT6 cells exhibited red fluorescence, indicating the efficient NIR‐II cell‐killing capability of the CQDs (Figure 3f). These results establish a biosafe basis for further NIR‐II photo‐thermoelectric therapy studies in mice.

### 2.4 STEs of CQD‐Triggered Pyroptosis

**Figure 4:**
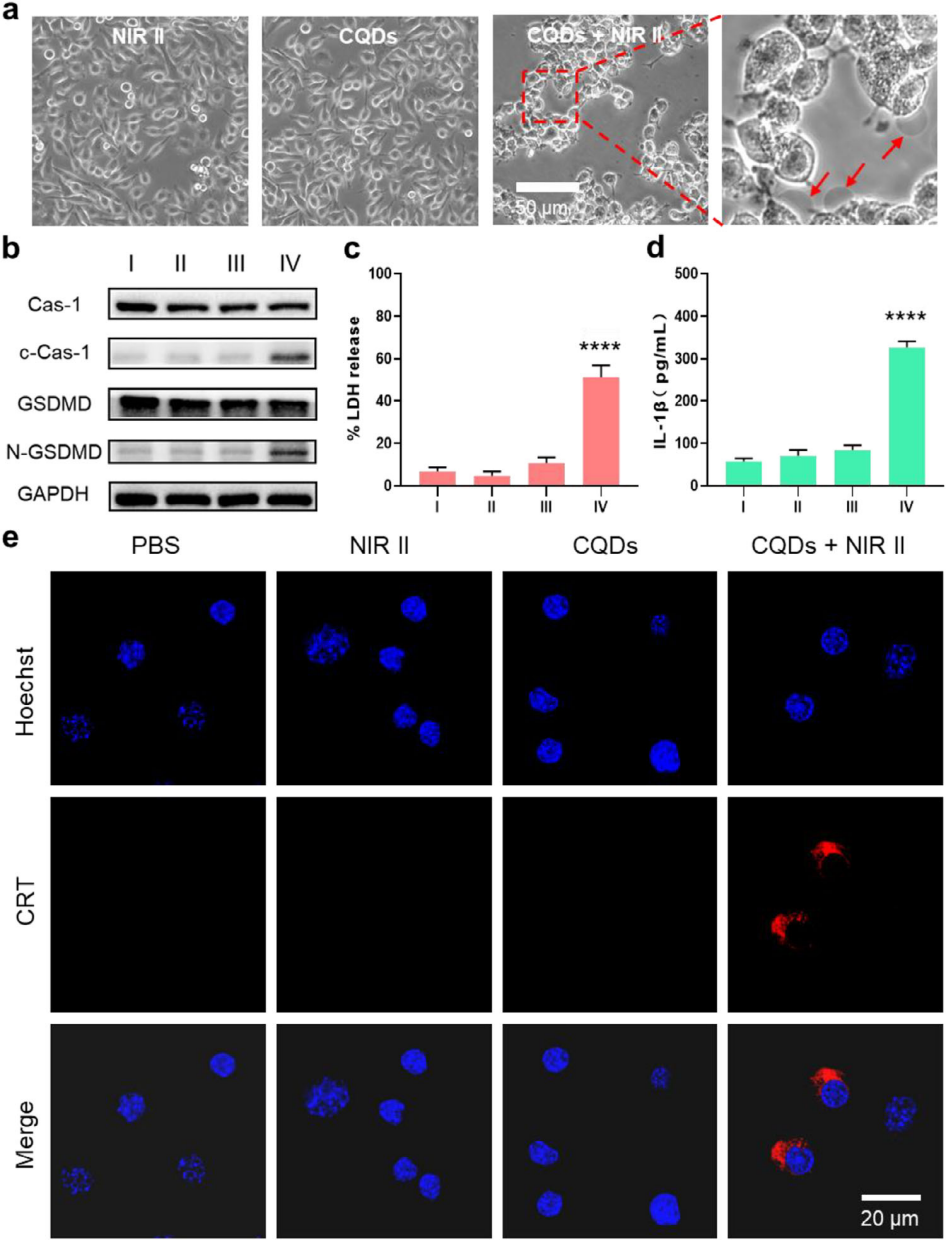
STEs of CQD‐triggered pyroptosis. a) Phase‐contrast imaging assay of CQDs triggered pyroptosis in EMT6 cells (arrows represent pyroptotic cells). b) Immunoblotting demonstrates caspase‐1 cleavage of GSDMD. c‐Cas1, Cleaved Caspase‐1; GSDMD‐F, full‐length GSDMD; GSDMD‐N, the N‐terminal cleavage products of GSDMD, respectively. c) LDH and d) IL‐1β release of the EMT6 cells treated with PBS (group I), NIR‐II light (group II), CQDs in the dark (group III), and CQDs after NIR‐II irradiation (group IV) (CQDs: 500 µg mL^−1^, NIR‐II: 0.8 W cm^−2^, 10 min). e) CLSM images of surface‐exposed CRT on EMT6 cells in different treatments (CQDs: 500 µg mL^−1^, NIR‐II: 0.8 W cm^−2^, 10 min). CRT: Calreticulin.

We conducted a phase‐contrast imaging assay in EMT6 cells via different treatments to reveal the STEs of CQDs‐triggered pyroptosis (**Figure** **4**a; Figure S8, Supporting Information). Compared with other control groups, nearly all EMT6 cells treated with the CQDs after NIR‐II light irradiation (group IV) were swollen with large bubbles from the plasma membrane, indicating highly efficacious pyroptotic behaviors. The GSDM family proteins are characteristic biomarkers for identifying the occurrence of pyroptosis. The overexpression of N‐domains of gasdermin D (N‐GSDMD) cleaved by cleaved caspase‐1 (c‐Cas1), has been demonstrated to be capable of eliciting pyroptosis. In group IV, c‐Cas1 and N‐GSDMD were overexpressed (Figure 4b), while other groups showed no apparent expression of the N‐GSDMD, suggesting that the STEs *of* CQDs triggered pyroptosis via the classical c‐cas1‐GSDMD pathway.

Then we quantitatively assessed the expression of three ICD indicators, such as LDH, IL‐1β, and CRT, to reveal the immunogenic form. As illustrated in Figure 4c,d, group IV exhibited a notable elevation in LDH and IL‐1β levels in the EMT6 cell supernatant (Figure S9, Supporting Information). In contrast, the remaining groups displayed insignificant changes in LDH and IL‐1β concentrations, signifying the essentiality and effectiveness of CQDs in inducing pyroptosis under NIR‐II irradiation. These findings were further validated through confocal laser scanning microscopy (Figure 4e), where the distinct red fluorescent signal of calreticulin on the cell membrane surface was exclusively observed in group IV. These prominent indicators of ICD unequivocally showcase the attributes of CQD‐induced pyroptosis in cancer cells, characterized by a highly immunogenic form of cellular demise. The release of a plethora of damage‐associated molecular patterns, including CRT, suggests that CQD‐induced pyroptosis in cancer cells holds the potential to instigate both innate and adaptive immune responses. Extensive evidence confirms that thermal energy alone preferentially induces apoptosis, not pyroptosis. Given the established scientific consensus that pyroptosis requires ROS activation, we conclude that ROS generated through thermoelectric catalysis processes serves as the principal trigger for pyroptosis in our system. Based on these, we propose that thermoelectric catalysis processes elevate oxidative stress levels, thereby stimulating mitochondrial transmembrane inflammasomes or translocases activation. This cascade triggers the caspase family proteins central to pyroptosis, primarily through the ROS‐NLRP3‐caspase‐1‐GSDMD signaling axis.^[^ ^20a^ ^]^ These results provide theoretical feasibility for our subsequent immunotherapy experiments.

### 2.5 NIR‐II Photo‐Thermoelectric Therapy and Immunotherapy

**Figure 5:**
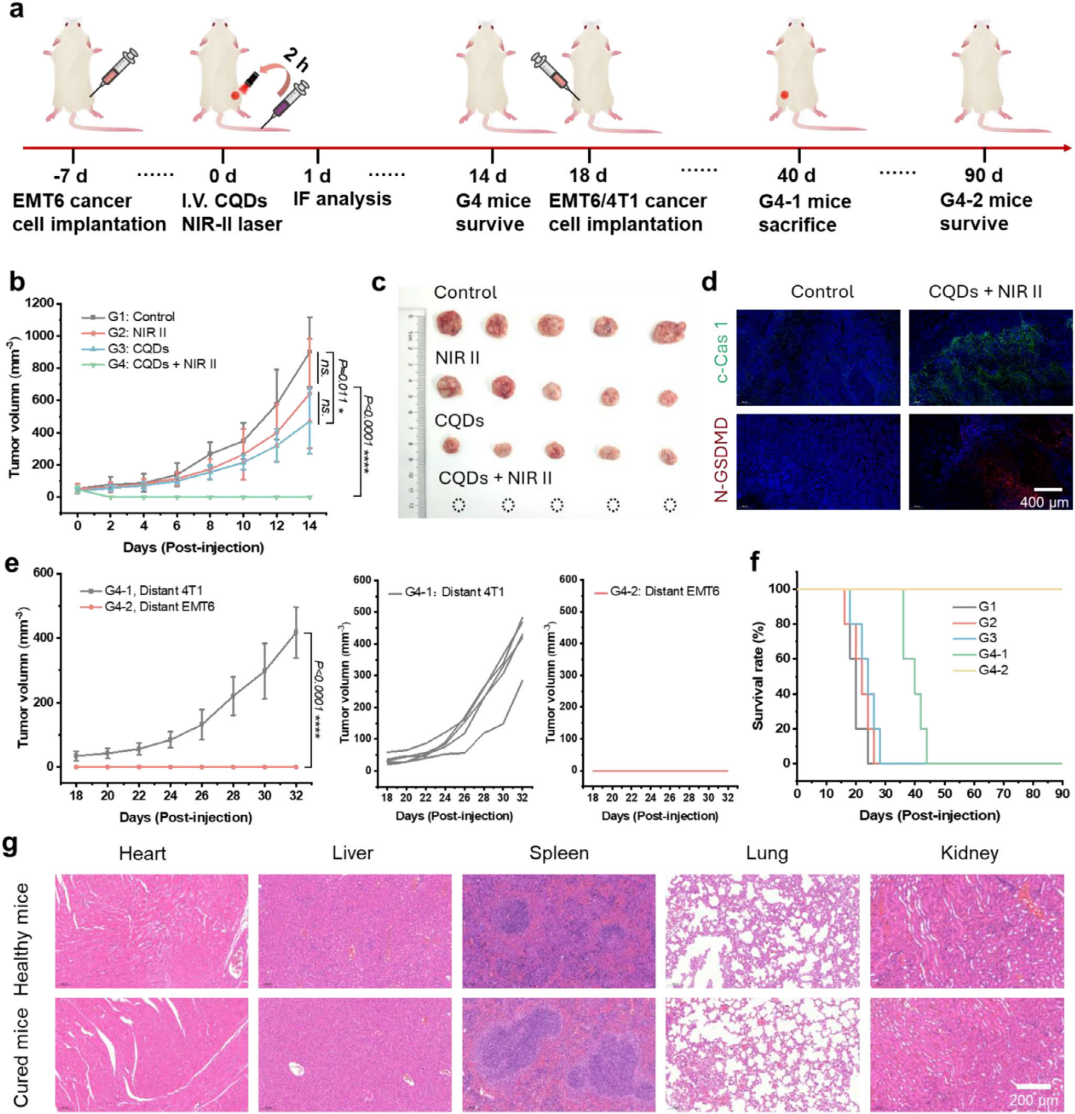
NIR‐II photo‐thermoelectric therapy and immunotherapy. a) Schematic illustration of the animal experimental design for cancer therapy. b) Growth curves of tumors from mice after different treatments (*n* = 5 for each group); the calculation was based on the mean tumor size (mean ± SD). c) Tumor images collected on day 14 (*n* = 5 for each group). d) c‐Cas1 and N‐GSDMD in tumors (Scale bar: 400 µm) of G1 and G3 (blue channel: 433–468 nm, Ex: 405 nm; red channel: 500–530 nm, Ex:488 nm). e) Tumor growth curves of G4‐1, G4‐2 on corresponding days; the calculation was based on the mean tumor size (mean ± SD). f) Survival rates of G1, G2, G3, G4‐1 and G4‐2 mice. g) H&E staining of major organs in G4 mice after 90 days compared with those in healthy mice. (G1: intravenous injection of PBS, G2: intravenous injection of PBS +1064 nm laser irradiation, G3: intravenous injection of the CQDs, G4: intravenous injection of the CQDs + 1064 nm laser irradiation, G4‐1: G4 group was reimplanted with 4T1 cancer cells, G4‐2: G4 group was reimplanted with EMT6 cancer cells).

The feasibility of utilizing the CQDs for tumor NIR‐II photo‐thermoelectric therapy and immunotherapy was further investigated. Forty‐eight mice bearing EMT6 tumors were randomly divided into four groups. When the tumor volume reached ≈50 mm^3^, the mice were intravenously injected with 100 µL of phosphate‐buffered saline (PBS) or the CQDs and subsequently kept in darkness or subjected to 1064 nm laser irradiation at 0.8 W cm^−2^ for 10 min. The groups consisted of mice injected with PBS without laser irradiation (G1), mice injected with PBS with laser irradiation (G2), mice injected with the CQDs (100 µL, 1000 µg mL^−1^) without laser irradiation (G3), and mice injected with the CQDs followed by laser irradiation (G4) (**Figure** **5**a). Remarkably, G4 achieved complete tumor eradication within 14 days post‐treatment, outperforming partial suppression in G3 and negligible effects in controls (Figure 5b,c; Figure S10, Supporting Information). This dramatic efficacy stems from the precisely engineered thermoelectric potential generated by the tumor‐localized thermal gradient, enabling targeted hydroxyl radical generation through thermoelectric catalytic effect. Immunofluorescence (IF) analysis of tumor tissues from two mice per group on Day 1 revealed elevated c‐Cas1 levels and increased N‐GSDMD expression in G4 tumors, validating pyroptosis activation through the canonical c‐Cas1/GSDMD axis (Figure 5d; Figure S11, Supporting Information). These results demonstrate that CQDs can function as both NIR‐II phototherapeutic agents and pyroptosis inducers. The therapeutic efficacy stems from the combined effects of thermoelectric catalytic ablation and the self‐cascading effect of pyroptosis. This work offers a promising novel direction for the future development of thermoelectric nanomaterials.

To decipher immunological memory, we rechallenged cured mice (*n* = 5) with either 4T1 (G4‐1) or EMT6 (G4‐2) cells. Strikingly, G4‐2 maintained 100% tumor‐free survival over 90 days, while G4‐1 developed progressive tumors (Figure 5e,f), demonstrating antigen‐specific immunological memory through pyroptosis‐released DAMPs. Lymphoid compartment profiling demonstrated treatment‐specific systemic immune activation, evidenced by concurrent CD4⁺ and CD8⁺ T‐cell infiltration in G4 and G4‐2 groups (Figures S12 and S13, Supporting Information). This coordinated T‐lymphocyte mobilization indicates robust abscopal immunomodulation. No discernible damage was observed in the organs of the mice treated with G4‐2 after day 90, showcasing similar tissue morphology to that of healthy mice, which verified the excellent biocompatibility of the CQDs (Figure 5g). These findings not only confirm the abscopal effect of localized pyroptosis induction but also establish STEs of CQDs as a clinical‐translatable platform for precision NIR‐II photo‐thermoelectric catalysis therapy and pyroptosis‐triggering immunotherapy.

## 3 Discussion

The strategic design of biosafe PTAs capable of synergizing deep‐tissue penetration with immunomodulation represents a critical advancement in precision oncology. Our work establishes a paradigm‐shifting methodology that harnesses the NIR‐II biowindow to simultaneously achieve tumor‐specific thermoelectric catalysis and pyroptosis‐mediated systemic immunity. Through rational engineering of CQDs with ultrahigh STE density, we realized an unprecedented NIR‐II photothermal conversion of 51.2% coupled with precisely tunable thermoelectric potentials. This breakthrough enables spatiotemporal control over ROS generation via temperature‐gradient‐driven charge separation, effectively overcoming the constraints of conventional single semiconductor bandgap and penetration‐immunity trade‐off that plagues conventional phototherapy. Mechanistically, we delineate that STE‐mediated pyroptosis activates a self‐amplifying immunogenic cascade. Gasdermin D pore formation triggers LDH, IL‐1β, and CRT et al release, resulting in complete primary tumor eradication and antigen‐specific recurrence prevention. These insights resolve the long‐standing paradox between localized ablation and systemic immune activation.

Our findings transcend conventional nanomaterial design principles by establishing STE as a universal NIR‐II absorber for thermoelectric‐immunotherapeutic performance. This conceptual framework opens avenues for developing “immuno‐photocatalysts,” while the demonstrated safety profile and manufacturing scalability position CQDs as clinically translatable pyroptosis inducers. The STE‐engineered NIR‐II photo‐thermoelectric therapy strategy represents a paradigm shift beyond conventional photothermal therapy and immune checkpoint blockade. Unlike traditional photothermal agents that rely on cytotoxic high temperatures for ablation,^[^ ^22^ ^]^ our platform leverages unique thermoelectric catalysis (enabled by high‐density STEs) to trigger pyroptosis at mild hyperthermia. This mechanism not only achieves efficient tumor suppression but also actively converts immunologically “cold” tumors into “hot” microenvironments via massive release of DAMPs, generating an in‐situ vaccination effect.^[^ ^23^ ^]^ A capability is absents in both conventional photothermal agents (limited to thermal killing) and immune checkpoint inhibitors (ICIs, dependent on pre‐existing tumor‐infiltrating lymphocytes). While challenges remain (e.g., material complexity, depth limitations shared with phototherapies, and potential risk of immune‐related adverse events similar to ICIs), this synergistic integration of NIR‐II drives thermoelectric catalysis and pyroptosis induction offers a promising, complementary approach with broader applicability potential, particularly for ICI‐resistant “cold” tumors. Future exploration of STE‐engineered materials may unlock unprecedented synergies between physical energy conversion and cancer immunology, ultimately providing a feasible solution for the metastatic malignancies therapy.

## 4 Experimental Section

Detailed experimental materials and methods can be found in the Supporting Information. All animal experiments were approved by the Fuzhou University Affiliated Provincial Hospital Animal Ethics Committee (protocol no. IACUC‐FPH‐SL‐20250207[0570]).

## Conflict of Interest

The authors declare that they have no competing interests.

## Author Contributions

T.Z., Q.W., Q.C., and H.W. contributed equally to this work. G.C. and T.Z. planned and supervised the project. G.C., T.Z., R.W., and Z.F. designed the overall studies, participated in the analysis of the results. T.Z. prepared the materials, carried out the spectroscopic measurements, and drafted the manuscript. Q.W., Q.C., H.F., H.Z., and M.L. assisted in the cell experiment. R.W., Z.F., H.W., H.Z., and F.C. assisted with bioimaging and phototherapy in vitro and in vivo. All the authors reviewed the manuscript.

## Supporting information



Supporting Information

## Data Availability

The data that support the findings of this study are available from the corresponding author upon reasonable request.

## References

[1] a) X. Li , J. F. Lovell , J. Yoon , X. Chen , Nat. Rev. Clin. Oncol. 2020, 17, 657; 32699309 10.1038/s41571-020-0410-2

[2] a) S. Shen , J. C. Qiu , D. Huo , Y. A. Xia , Small 2024, 20, 2305426; 10.1002/smll.202305426 PMC10922052 37803412

[3] D. F. Quail , J. A. Joyce , Nat. Med. 2013, 19, 1423. 24202395 10.1038/nm.3394 PMC3954707

[4] D. J. Irvine , E. L. Dane , Nat. Rev. Immunol. 2020, 20, 321. 32005979 10.1038/s41577-019-0269-6 PMC7536618

[5] a) M. Ma , Y. Zhang , K. Pu , W. Tang , Chem. Soc. Rev. 2025, 54, 653; 39620588 10.1039/d4cs00679h

[6] A. I. Birmpilis , A. Paschalis , A. Mourkakis , P. Christodoulou , I. Kostopoulos , E. Antimissari , G. Terzoudi , A. G. Georgakilas , C. Armpilia , P. Papageorgis , E. Kastritis , E. Terpos , M. A. Dimopoulos , H. Kalbacher , E. Livaniou , M. I. Christodoulou , O. E. Tsitsilonis , Cells‐Basel 2022, 11, 1415. 10.3390/cells11091415 PMC9102069 35563721

[7] B. T. Cookson , M. A. Brennan , Trends Microbiol. 2001, 9, 113. 11303500 10.1016/s0966-842x(00)01936-3

[8] a) L. L. Chen , X. Ma , W. J. Liu , Q. Q. Hu , H. H. Yang , Nano Lett. 2023, 23, 8725; 37695255 10.1021/acs.nanolett.3c02728

[9] a) P. Broz , P. Pelegrín , F. Shao , Nat. Rev. Immunol. 2020, 20, 143; 31690840 10.1038/s41577-019-0228-2

[10] a) H. Wang , T. Wang , S. Yan , J. Tang , Y. Zhang , L. Wang , H. Xu , C. Tu , Mol. Cancer 2024, 23, 268; 39614288 10.1186/s12943-024-02183-9 PMC11607834

[11] a) Z. D. Chen , G. Xu , D. Wu , S. H. Wu , L. Gong , Z. H. Li , G. H. Luo , J. Hu , J. Chen , X. T. Huang , C. C. Chen , Z. Y. Jiang , X. M. Li , Biochem. Pharmacol. 2020, 177, 114023; 32413426 10.1016/j.bcp.2020.114023

[12] a) Y. N. Liu , T. Zeng , S. Y. Bai , X. Fang , X. P. Cao , S. Q. Li , Q. Chen , C. H. Lu , H. H. Yang , Adv. Funct. Mater. 2025, 2505784;

[13] a) M. Wu , X. G. Liu , H. Chen , Y. K. Duan , J. J. Liu , Y. T. Pan , B. Liu , Angew. Chem. Int. Ed. 2021, 60, 9093; 10.1002/anie.202016399 33543534

[14] S. Zeng , J. Y. Wang , H. Kang , H. D. Li , X. J. Peng , J. Y. Yoon , Angew. Chem. Int. Ed. 2025, 137, 202417899. 10.1002/anie.202417899 39513509

[15] a) L. Manna , T. W. Odom , Proc. Natl. Acad. Sci. USA 2024, 121, 2410357121; 10.1073/pnas.2410357121 PMC11260145 38985756

[16] a) B. Y. Wang , S. Y. Lu , Matter‐Us 2022, 5, 110;

[17] a) T. S. Zhang , B. Z. Wang , Q. S. Cheng , Q. C. Wang , Q. Q. Zhou , L. Y. Li , S. N. Qu , H. D. Sun , C. X. Deng , Z. K. Tang , Sci. Adv. 2024, 10, adn7896; 10.1126/sciadv.adn7896 PMC11225785 38968361

[18] T. S. Zhang , Q. S. Cheng , H. W. Cheng , Q. C. Wang , B. Z. Wang , B. H. Zhang , H. D. Sun , C. X. Deng , Z. K. Tang , Nat. Commun. 2025, 638, E16.

[19] a) Z. C. Deng , Y. J. Zhang , R. Q. Li , Y. Y. Zhu , C. X. Xu , B. W. Gao , W. L. Wang , C. G. Ding , B. He , X. Z. Zhu , M. Yang , T. Liang , M. Z. Zhang , Adv. Funct. Mater. 2025, 35, 2418683;

[20] a) Q. S. Cheng , T. S. Zhang , Q. C. Wang , X. Wu , L. Y. Li , R. X. Lin , Y. N. Zhou , S. N. Qu , Adv. Mater. 2024, 36, 2408685; 10.1002/adma.202408685 39129656

[21] G. Zoppellaro , M. Medved , V. Hruby , R. Zboril , M. Otyepka , P. Lazar , J. Am. Chem. Soc. 2024, 146, 15010. 38696712 10.1021/jacs.3c13296 PMC11157526

[22] Y. L. Song , Y. L. Wang , Y. Zhu , Y. Cheng , Y. D. Wang , S. Y. Wang , F. P. Tan , F. Lian , N. Li , Adv. Healthcare Mater. 2019, 33, 2007488.

[23] a) Z. C. Wang , Y. Q. Tang , Q. Li , Light‐Sci. Appl. 2025, 14, 16; 39743555 10.1038/s41377-024-01673-1 PMC11693763

