# Supplementary material for: Self‐Trapped Excitons in Carbon Quantum Dots with Large NIR‐II Photo‐Thermoelectric Catalysis Induce Pyroptosis for Cancer Therapy
Source: Adv Sci (Weinh). 2025 Aug 22;13(4):e14249. doi: 10.1002/advs.202514249 (PMC12822447; doi:10.1002/advs.202514249)
Supplement: Supplementary file 1 — Supporting Information [file ADVS-13-e14249-s001.docx]

**Supplementary Information for:**

**Self-trapped excitons in carbon quantum dots with large** **NIR-II** **photo-thermoelectric catalysis induce** **pyroptosis for precision cancer therapy**

Tesen Zhang^1, 4, #,^ *, Qingcheng Wang^4,^ ^#^, Quansheng Cheng^4, #^, Han Wang^5, #^, He Feng^4^, Hui Zhang^2^, Mengbo Lin^2^, Feili Cai^2^, Zheng Fang^6,^ *, Ruo Wang^2,^ *, Gang Chen^3,^ *

*^1^ Interdisciplinary Institute of Medical Engineering, Fuzhou University, Fuzhou, 350108, China*

*^2^ Shengli Clinical Medical College of Fujian Medical University, Department of Breast Surgery, Fujian Provincial Hospital, Fuzhou University Affiliated Provincial Hospital, Fuzhou University, Fuzhou, 350001, China*

*^3^ Shengli Clinical Medical College of Fujian Medical University, Department of Endocrinology, Fujian Provincial Hospital, Fuzhou University Affiliated Provincial Hospital, Fuzhou, 350001, China*

*^4^ Institute of Applied Physics and Materials Engineering, University of Macau, Taipa, Macau SAR, China*

*^5^ Department of General Surgery, Comprehensive Breast Health Center, Ruijin Hospital, Shanghai Jiao Tong University School of Medicine, Shanghai, China*

*^6^ Institute of Molecular Medicine, Renji Hospital, School of Medicine, Shanghai Jiao Tong University, Shanghai 200127, China*

E-mails: yc17811@um.edu.mo, chengangfj@163.com, wangruo@fzu.edu.cn, zhengfang2021@sjtu.edu.cn

Contents

[Methods 3](#_Toc154707662)

[Chemical structures of the CQDs. 11](#_Toc154707662)

[Lattice structures of the CQDs. 12](#_Toc154707663)

[NIR II photothermal performance 13](#_Toc154707664)

[Optical property of CQDs 15](#_Toc154707665)

[Distribution of CQDs in biological systems. 18](#_Toc154707665)

[Morphology of CQDs in biological environment. 19](#_Toc154707665)

[STEs of CQD-triggered pyroptosis 20](#_Toc154707665)

[LDH release of the EMT6 cells.. 21](#_Toc154707667)

[NIR-II cancer therapy. 22](#_Toc154707667)

[Reference 26](#_Toc154707674)

**Methods**

**Materials.** Citric acid, urea, formic acid, ethanol, and DMSO were used. Water was deionized and purified. All reagents had at least analytical grade purity and were used as received without further purification. All animal experiments were approved by the Fuzhou University Affiliated Provincial Hospital Animal Ethics Committee (protocol no. IACUC-FPH-SL-20250207[0570]). According to the guidelines, mice were sacrificed when the largest tumor size limit was 1.5 cm (at the largest dimension), 1,500 mm^3^ (for a subcutaneous tumor model) or a weight loss of 20% (for an orthotopic tumor model).

**Sample preparation.** For the CQDs, 1 g of citric acid and 2 g of urea were placed in a Teflon autoclave with 10 mL formic acid and then subjected to a constant temperature over 180 °C for a few hours. The resulting dark brown solution was combined with ethanol and centrifuged to eliminate any residual impurities. The resulting precipitate was redissolved in water and dialyzed against deionized water using 1000 Da dialysis bags for 24 h. The solution in the dialysis bags was collected and freeze-dried to obtain the CQDs.

**Characterization.** TEM images were captured using an FEI Tecnai-G2-F30 electron microscope with an acceleration voltage of 200 kV. AFM measurements were performed using a BRUKER ICON instrument. Raman spectra were acquired using a Horiba LABHRev-UV Raman spectrometer. Ultraviolet–visible absorption spectra were recorded on a JASCO V-770 spectrophotometer, and PL spectra were obtained using an Ocean Optics QE Pro spectrofluorometer. Femtosecond TA spectra of various samples were collected using an Ultrafast Systems HELIOS TA spectrometer. The laser source utilized was the Coherent Astrella-1K-F Ultrafast Ti: Sapphire Amplifier (800 nm, 1 kHz, <100 fs). The broadband probe pulses were generated by focusing a small portion of the fundamental 800 nm laser pulses into an Al_2_O_3_ plate. The 550 nm and 700 nm pump pulses were generated by a Light Conversion TOPAS-C optical parametric amplifier. Electron paramagnetic resonance (EPR) spectroscopy was conducted on a Magnettech ESR5000 (Bruker) instrument. The light source for these measurements was lasers, the NIR-I source was a 690 nm laser (1 W cm^−2^), and the NIR-II source was a 1064 nm laser (0.8 W cm^−2^). XPS analyses were conducted by an ESCALAB 250Xi spectrometer (Thermo Fisher Scientific, USA) with the monochromatic X-ray source (Al K_α_ hν = 1486.7 eV) in an ultrahigh-vacuum (UHV) chamber (base pressure, 5.0 × 10^−10^ mbar). Raman spectra were acquired using a Horiba LABHRev-UV Raman spectrometer.

**Electron paramagnetic resonance (EPR) test.** DMPO was used as a spin trap agent to measure the production of hydroxyl radical in water and superoxide radical in DMSO solution. CQDs were dissolved in water and DMSO at the concentration of 25 μg mL^-1^, then 1% DMPO was added and mixed well. The mixture was sucked up by a capillary tube, sealed with clay and irradiated under NIR-II light (0.8 W cm^-2^). At different time intervals, the EPR spectroscopy was applied to detect the EPR signals. The EPR signal of DMPO alone at different time intervals was also measured under the same conditions as control. The signal of spin electrons was also measured by EPR spectroscopy. CQDs were dissolved in aqueous solution at the concentration of 25 μg mL^-1^. The solution was sucked up by a capillary tube, sealed with clay and irradiated under NIR-II light (0.8 W cm^-2^). At different time intervals, the EPR spectroscopy was applied to detect the EPR signals.

**Cell culture.** Mouse breast cancer 4T1 and EMT6 cells were cultured in Roswell Park Memorial Institute 1640 medium (GIBCO), supplemented with 10% fetal bovine serum (GIBCO) and 100 U mL^−1^ penicillin and 100 µg mL^−1^ streptomycin (GIBCO). Human Cardiomyocyte AC16 cells were maintained in Dulbecco’s modified eagle medium (GIBCO), supplemented with 10% fetal bovine serum (GIBCO) and 100 U mL^−1^ penicillin and 100 µg mL^−1^ streptomycin (GIBCO). All cells were cultured in a humidified incubator at 37°C with 5% CO_2_.

**Cell viability assay (CCK-8 assay).** The CCK-8 assay was also conducted to evaluate the cytotoxicity of the CQDs to 4T1, EMT6, and AC16 cells. First, ~5000 cells were seeded in 96-well plates and incubated for 12 h. Subsequently, the medium was replaced with 100 μl of fresh medium containing varying concentrations of the CQDs. After 48 h of incubation, the cells were treated with CCK-8 reagent for 3 h before the absorbance at 450 nm was measured using a Tecan microplate reader (Thermo Scientific).

**Imaging of pyroptotic cells**. Briefly, EMT6 cells were seeded onto 6 well plates for 24 h, respectively. The cells in the different groups were then subjected to the following treatments: Group I, incubated with PBS; Group II, NIR-II light, 0.8 W cm^-2^, 10 min; Group III, incubated with 500 µg mL^−1^ CQDs; Group IV, incubated with 500 µg mL^−1^ CQDs for 1h and irradiated with NIR-II light for 10 min at 0.8 W cm^-2^. The concentration of CQDs, as well as the duration and intensity of light exposure, are determined based on maximizing pyroptosis efficiency. Alternative durations or intensities may result in reduced or absent pyroptosis. After treatment, phase-contrast images were collected by Inverted microscope (CKX53, Olympus) and camera (JENOPTIK GRYPHAX^®^). A more specific experimental description for the swelling bubbles imaging: after the above treatment, the cells were slowly placed in the incubator. Then, they were observed under a microscope every half an hour. Once morphological changes are found, the cells were washed with PBS preheated at 37°C and then the images were collected immediately.

**Calcein AM and PI assay.** To evaluate the impact of the CQDs on EMT6 cells, approximately 1×10^5^ EMT6 cells were cultured overnight in glass-bottom dishes. Subsequently, the EMT6 cells were subjected to different conditions: incubation without the CQDs, incubation with the CQDs (500 µg mL^−1^) for 1 h, irradiation with a 1064 nm laser for 10 min, and staining with calcein acetoxymethyl ester (calcein AM) at 37°C for 30 min. After being washed thrice with PBS solution, the cells were observed using a fluorescence microscope (Olympus IX73 inverted microscope) with a 20× objective lens at 470 nm excitation and a 500 nm LP filter.

**Cytokine, LDH release measurement**. The concentrations of IL-1β in the various sample supernatants were determined using IL-1β ELISA kits (Beyotime Biotechnology, PI301). LDH was measured using the CytoTox96TM Non-Radioactive Cytotoxicity Assay Kit (Promega, USA). To normalize for spontaneous lysis, the percentage of LDH release was calculated as follows: (LDH treated - LDH untreated cells) / (LDH total lysis - LDH untreated cells) × 100. Plates were read at 450 nm using a Varioskan LUX Multimode Microplate Reader (Thermo Fisher Scientific). EMT6 cells were inoculated into 96-well plates at a density of 1 × 10^4^ cells per well and incubated overnight. Subsequently, a distinct formulation (CQDs: 500 μg mL^-1^) was added to each well, followed by incubation for 24 h, and then irradiated with a NIR-II light at 0.8 W cm^-2^ for 10 minutes.

**Intracellular Detection of CRT (Immunofluorescence, IF)**. EMT6 cells were seeded in a 24-well plate (slides or coverslips, inside) at a density of 3×10^4^ cells per well and were incubated overnight. The cells were then treated as follows: Group I, incubated with PBS; Group II, NIR-II light, 0.8 W cm^-2^, 10 min; Group III, incubated with 500 µg mL^−1^ CQDs; Group IV, incubated with 500 µg mL^−1^ CQDs for 1 h and irradiated with NIR-II light for 10 min at 0.8 W cm^-2^. The EMT6 cells were then fixed using 4% paraformaldehyde and permeabilized with 0.1% Triton X-100. Subsequently, the cells were incubated with anti-CRT antibodies (cell signaling, 12238) and a Cy3-conjugated anti-rabbit secondary antibody (Beyotime Biotechnology, A0516). The cells were counterstained with Hoechst 33258, and confocal laser scanning microscopy was utilized to detect the CRT fluorescence.

***In vivo* pharmacokinetics**. Five-week-old female BALB/c mice were used for pharmacokinetics studies with three mice for each experiment group. All mice were intravenously injected with CQDs at a dose of 20 mg kg^−1^. The blood was collected at needed time points using a capillary tube from the retro-orbital sinus. The concentration of CQDs in blood was estimated by fluorescent intensity using an InGaAs array detector. The excitation laser was a 589 nm laser diode at a power density of ~ 100 mW cm^−2^, and emission was collected with 650-nm long-pass filter. The percentage of the CQD in blood was calculated as

*%ID g^-1^* = (*F*_t_ – *F*_control_) / [ (*F_injuected_ – F_control_*) × *M*_t_] × *100%*

where *F_t_* is the fluorescent intensity of collected blood as measured with a 650-nm long-pass filter, *F*_control_ is the fluorescent intensity of control blood, *F*_injected_ is the fluorescent intensity of the injected CQD (mixed with blood), *M*_t_ is the mass of the collected blood.

**Biochemistry tests.** During the collection of data about *in vivo* biomechanical parameters, blood samples were also collected carefully from the central artery of each mouse’s ear using a 5-gauge needle. This procedure was repeated on days 0, 1, 15 and 60 days, with 3–5 ml of blood being collected each time. EDTA vacuum tubes were used for collection, and the blood was continuously agitated to prevent coagulation. The blood samples were then stored at 4 °C for further processing. Meanwhile, 1.5–2 ml of the serum was separated from the whole blood by centrifuging at 3000 rpm for 15 min and stored frozen at −20 °C for subsequent analysis. The blood samples were utilized for routine blood tests using Rayto Chemray 420, providing valuable insights into the mouse’ hematological status. While the serum samples were specifically earmarked using Mindray BC-2800vet for liver and kidney biochemistry function tests, enabling a comprehensive assessment of the mice’ physiological responses (*n* = 3 per group).

***In vivo* metabolism study.** For *in vivo* organ metabolism imaging of the CQD aqueous solution (1000 µg mL^−1^), 6–8-week-old female BALB/c mice were intravenously injected (via the tail vein) at different time points (0, 1, 3, 6, 12, and 24 h) before being sacrificed to obtain organ images. Images were obtained using a ChemDocTM MP Imaging System (Bio-Rad Laboratories, Inc.) with 589 nm excitation light and a 650/50 nm emission filter. No animals exhibited any signs of acute toxicological responses during the experiments.

**Photothermal effect measurements.** Photothermal effect data were obtained using UNT-T323 digital thermometers with a K-type thermocouple. A volume of 1 mL of the CQD aqueous solution (500 µg mL^−1^) was introduced into a quartz cuvette, which was then irradiated with a 1064 nm laser at a power density of 1 W cm^−2^ for 10 min. Pure water was used as a negative control. The thermocouple probe was connected to the digital thermometer and inserted into the CQD aqueous solution perpendicular to the light path. The temperature of the CQD aqueous solution was recorded at 30 s intervals using a digital thermometer. The change in temperature of the CQD aqueous solution as a function of time was monitored under 1064 nm laser irradiation until room temperature was reached. According to the obtained data, the PCE was calculated to be ~51%.

***In vivo* tumor NIR-II photo-thermoelectric therapy.** A tumor-bearing mouse model was established by subcutaneously injecting EMT6 cells (5 × 10^5^ per mouse) into the upper dorsal region of female BALB/c mice. Once the tumor volume reached ~70 mm3, the mice were randomly divided into four groups and intravenously injected with 100 μL of PBS or CQDs (1000 µg mL^−1^) (n = 5 per group) with and without a 1064 nm laser (0.8 W cm^−2^) irradiation for 10 min. Cured mice were divided into two groups and subsequently injected distally with 4T1 and EMT6 cells, respectively (n = 5 per group). The tumor volume was recorded every two days and calculated using the formula V = ((tumor length) × (tumor width)^2^)/2. Mice were sacrificed on day 16 after the initial drug treatment.

**Histopathological evaluation.** For histological analysis, the organs (heart, liver, spleen, lung, and kidney) were fixed in 10% formalin and then embedded in paraffin. Slices of these organs from the mice were stained with Hematoxylin & Eosin (H&E) and IF. The histological sections were imaged by an optical microscope.

**Statistical analysis.** The data were presented as the mean ± S.D. The statistical significance of differences among groups was assessed using the student’s t-test.


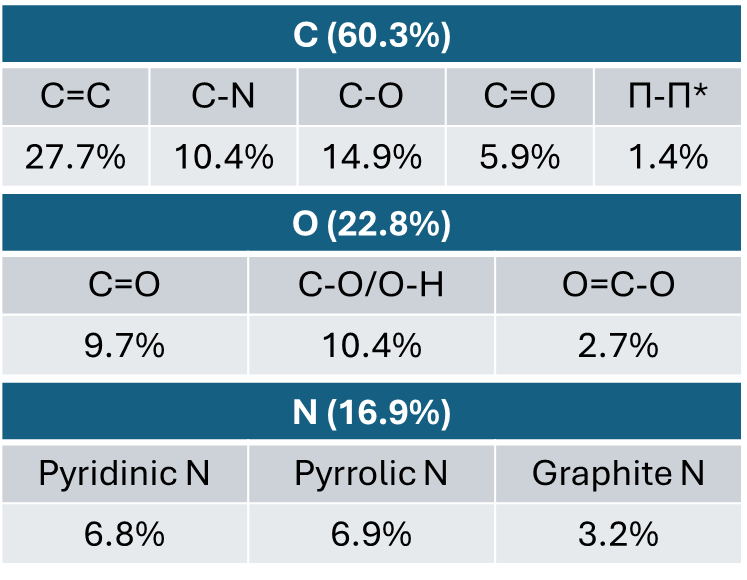


Table. S1. Chemical structures of the CQDs. Specific C, N and O content for different chemical bonds in CQDs in the XPS spectra

**Figure S1. Lattice structures of the CQDs.** Roman spectra of CQDs.


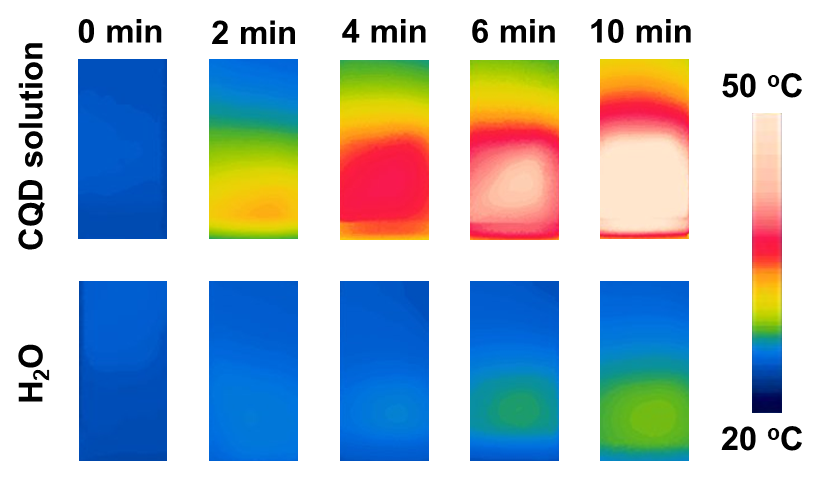


**Figure S2. NIR-II photothermal performance of the CQDs.** Photothermal images of the CQD aqueous solution at 500 μg ml^−1^ at various times under 1064-nm laser irradiation at 0.8 W cm^−2^.


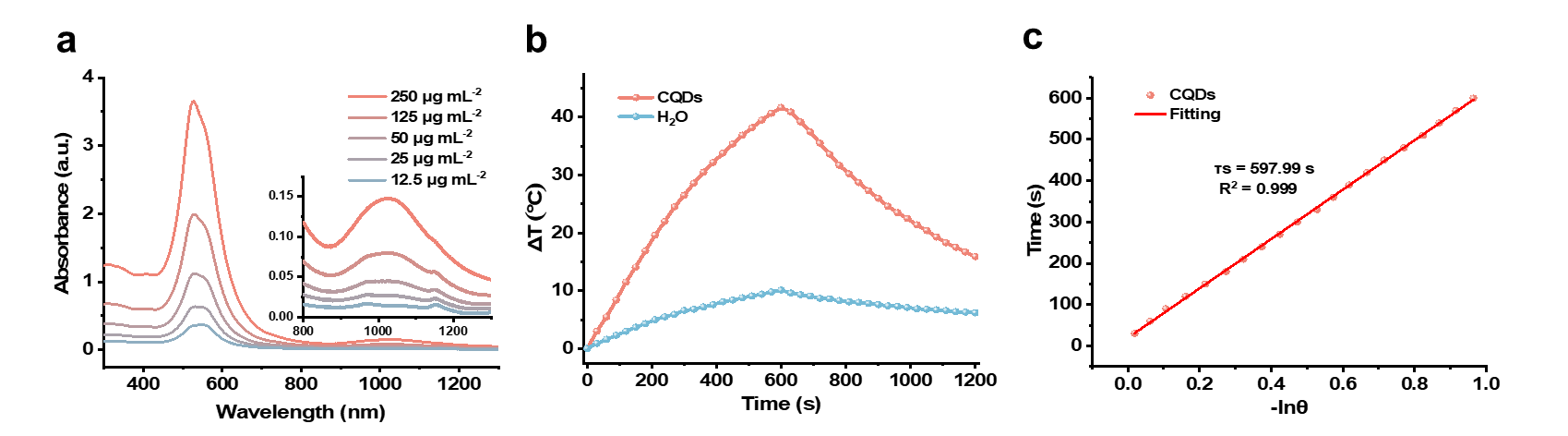


**Figure S3. NIR-II photothermal performance of the CQDs. a,** Absorbance of CQD solution at various concentrations. **b,** Temperature evolutions of CQD aqueous solutions (500 µg mL^−1^) and pure water under 1064 laser irradiation at 1 W cm^−2^; the laser was turned off after irradiation for 600 s. Time constants for heat transfer from the **c,** CQD systems were calculated as τ_s_ (CQDs) = 597.99 s by plotting the linear time data from the cooling period (after 600 s) against the negative natural logarithm of the driving force temperature obtained from the cooling stage (a).

The PCE (*η*) was calculated using the following equation^1^:

$\eta=\frac{hS(T_{\max}-T_{\mathrm{surr}})-Q_{\mathrm{Dis}}}{I(1-{10}^{-A_{1064}})}$(1)

where *h* (mW/(m^2^∙°C)) is the heat transfer coefficient, *S* (m^2^) is the surface area of the container, *T*_max_ is the equilibrium temperature, and *T*_surr_ is the ambient temperature of the surroundings. As shown in Figure S3a, *T*_max_−*T*_surr_ was 41.7 °C (CQDs) and 10.1°C (pure water). *Q*_Dis_ (mW) denotes the heat from light absorbed by the cuvette sample walls and was independently measured as 31.04 mW using a quartz cuvette cell containing aqueous samples without CQDs. *I* is the incident laser power (1 W), and *A*_1064_ is the absorbance of the CQDs (0.3106) at 1064 nm.

To calculate *hS*, *θ* is introduced using the maximum system temperature, *T*_max_

$\theta=\frac{T-T_{\mathrm{surr}}}{T_{\max}-T_{\mathrm{surr}}}$ (2)

The sample system time constant τ_s_ was determined as:

$\tau_{s}=\frac{\sum_{i} m_{i}C_{p,i}}{hS}$ (3)

According to the following expression

$t=-\tau_{s}ln(\theta)$ (4)

τ*_s_* was calculated as 597.99s (CQDs). Substituting *m* = 1 g and *C* = 4.2 J/g∙K in Eq. (3), the values for *hS* were determined as 7.02 mW/°C (CQDs). Finally, *η* was calculated from Eq. (1) as 51.22 % (CQDs)

**Figure S4. Optical property characterization.** EPR spectra of CQDs in DMSO solution after irradiation by NIR-II light for different temperatures.


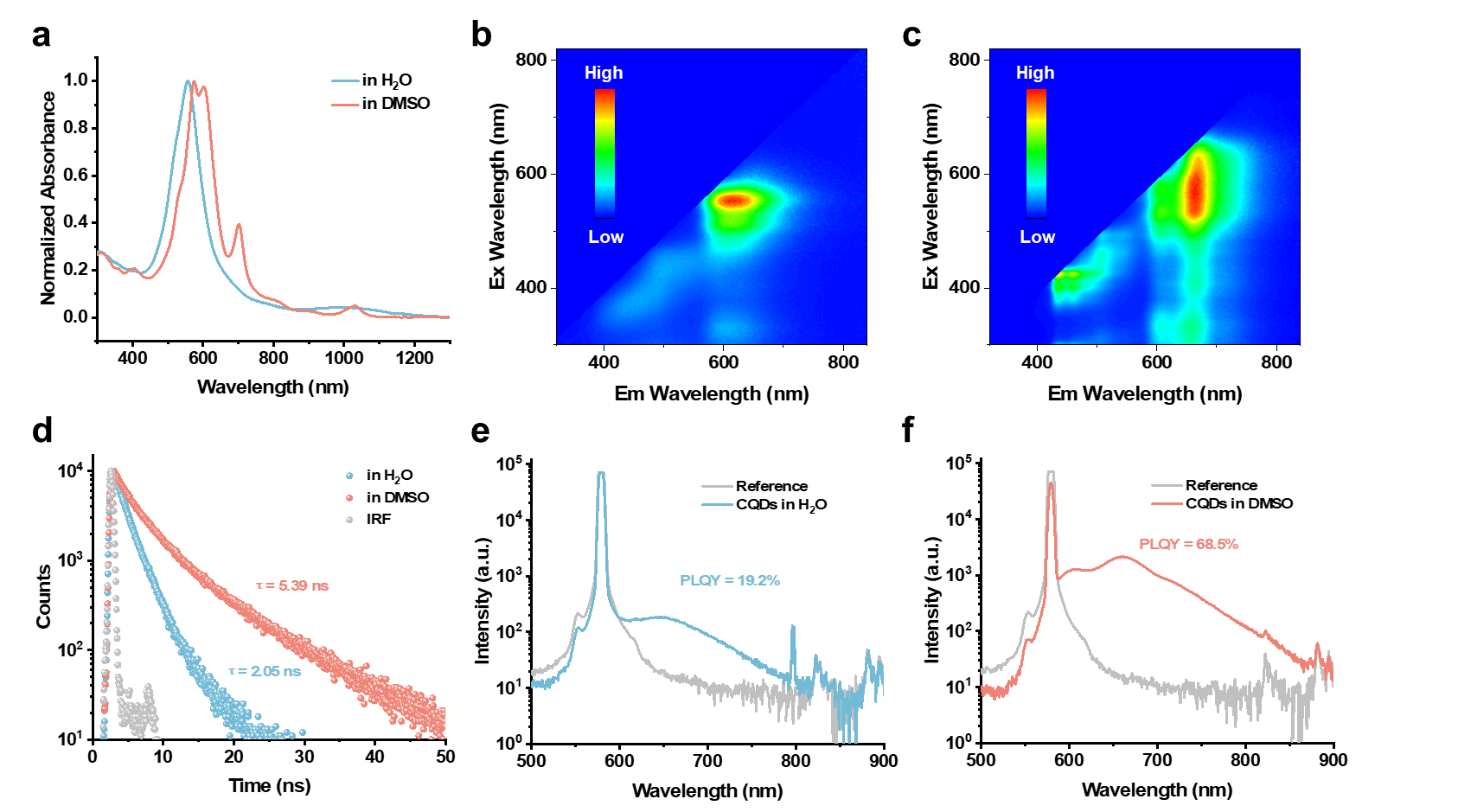


**Figure S5. Optical property characterization. a,** Normalized absorption spectra of CQD in aqueous solution and DMSO solution. Excitation-emission maps of CQDs in **b,** aqueous solution and **c,** DMSO solution. **d,** Luminescence decays of CQDs in water, in DMSO monitored at 640 nm under 510 nm excitation (IRF = instrument response function). Photoluminescence quantum yield of CQDs in **e,** water and **f,** DMSO solution.


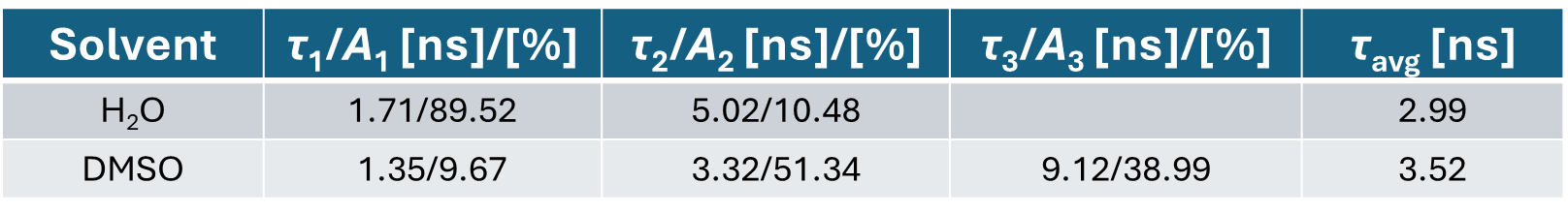


**Table. S2. Optical property characterization.** Fluorescence lifetimes of CQDs.


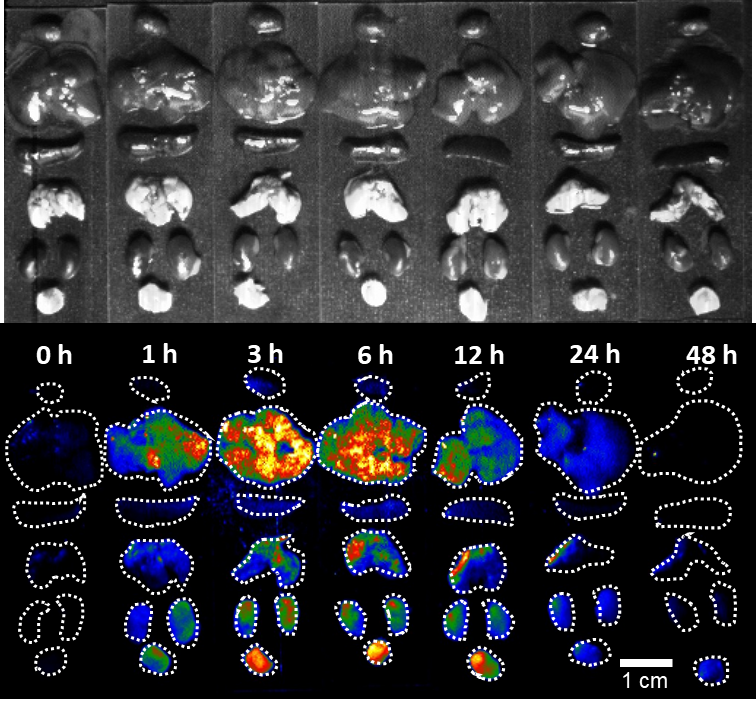


**Figure S6. Distribution of CQDs in biological systems.** Bright-field and fluorescence field photos of major mice organs at several time points before and after the intravenous injection of CQD aqueous solutions (100 µL, 1000 µg mL^−1^).


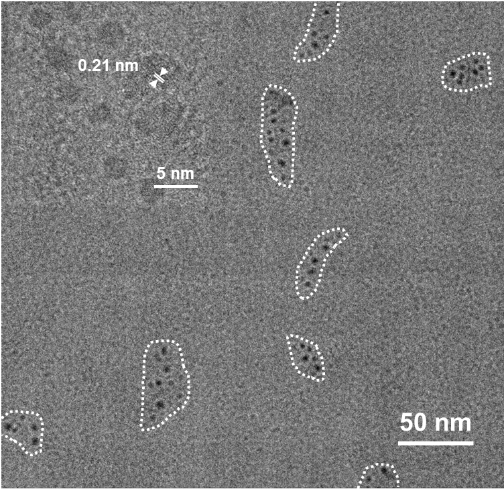


**Figure S7. Morphology of CQDs in biological environment.** TEM and HRTEM (inset) images of CQDs in serum.


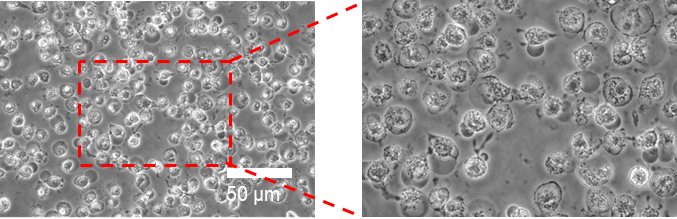


**Figure S8. STEs of CQD-triggered pyroptosis.** Phase-contrast imaging assay of CQDs triggered pyroptosis in EMT6 cells

**Figure S9. LDH release of the EMT6 cells^2^.** The standard curve of absorption intensity (450 nm) as a function of concentration of IL-1β in Enzyme linked immunosorbent assay (ELISA).


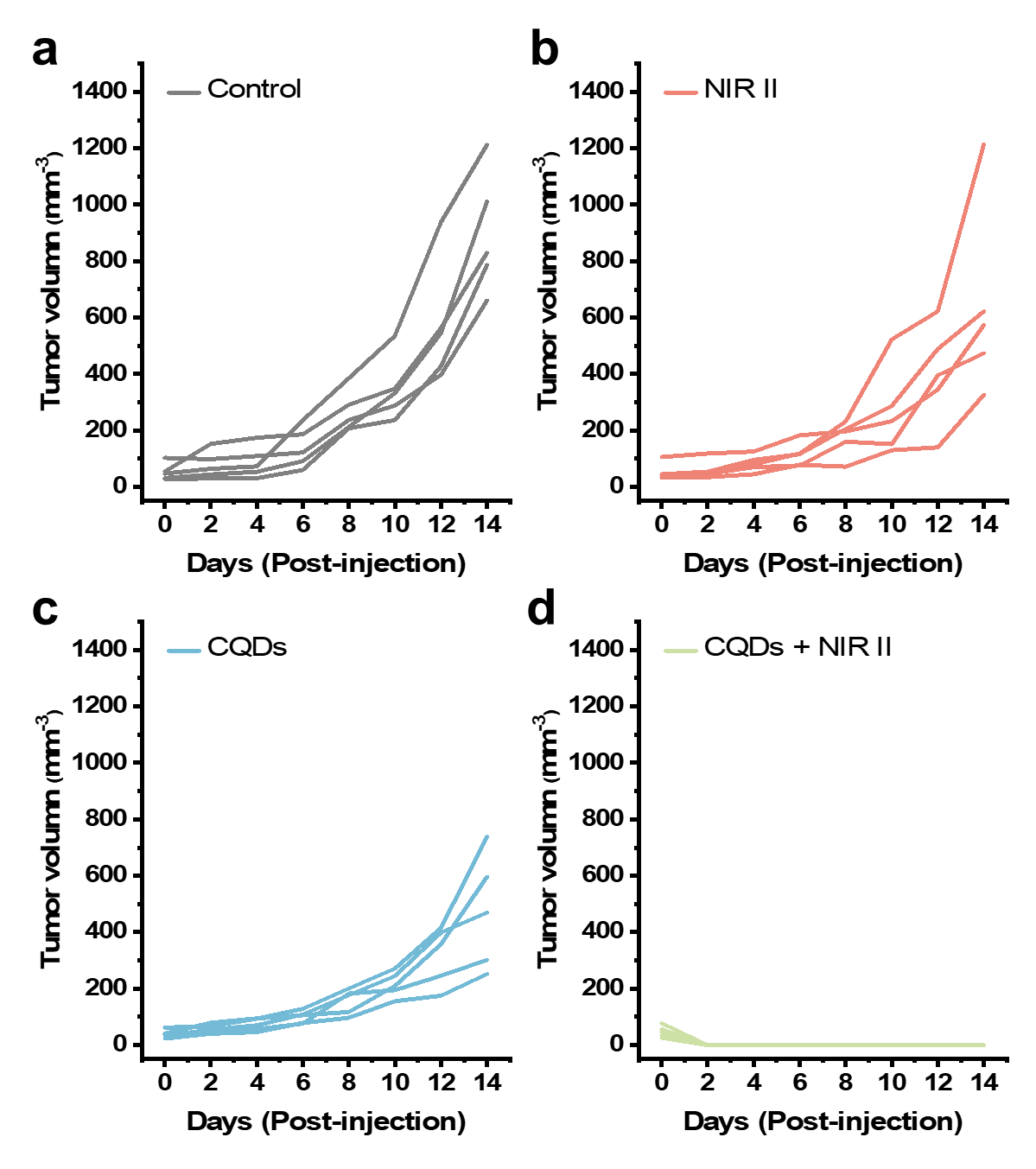


**Figure S10. NIR-II cancer therapy.** Tumor growth curves in G1-G4


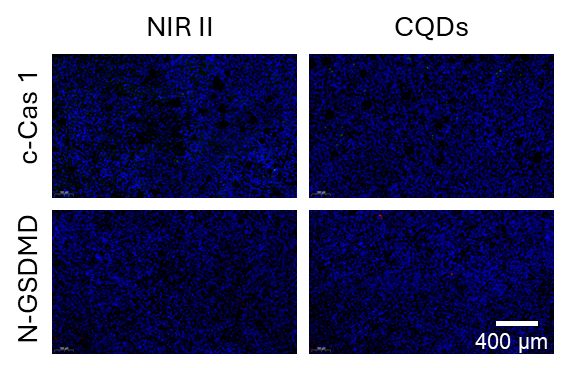


**Figure S11. NIR-II cancer therapy.** c-Cas1 and N-GSDMD in tumors (Scale bar: 400 µm) of G2 and G3 (blue channel: 433–468 nm, Ex: 405 nm; red channel: 500–530 nm, Ex:488 nm).


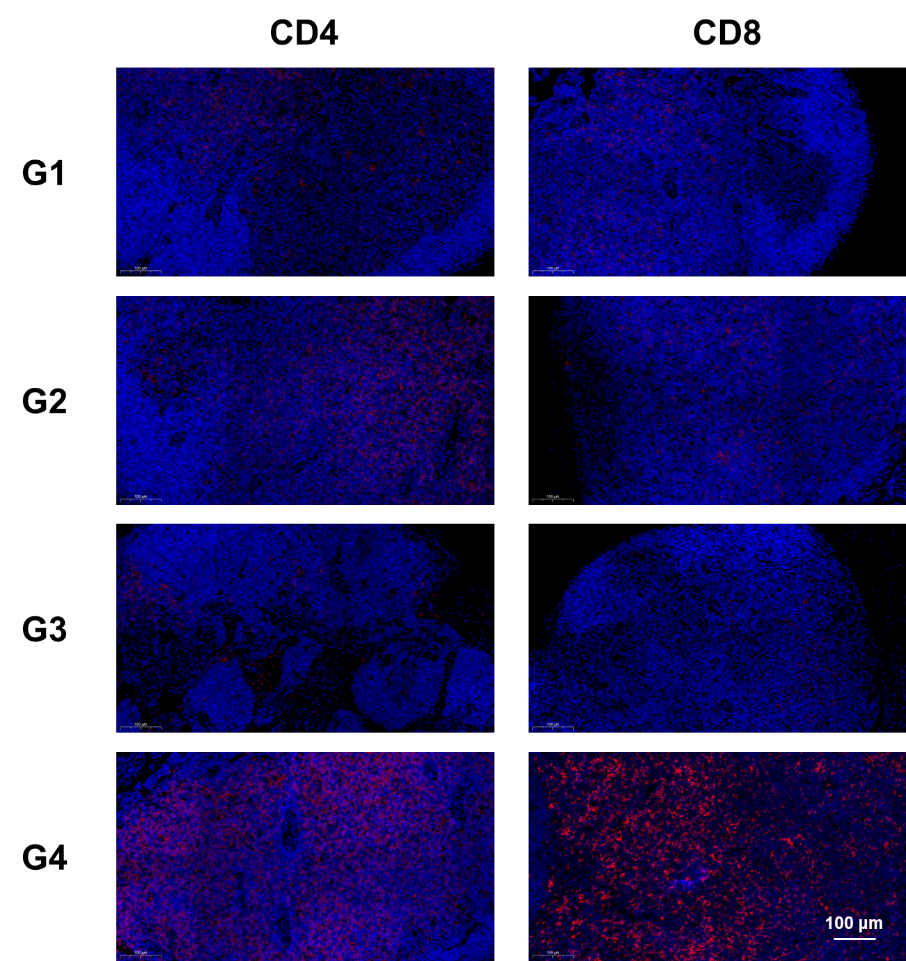


**Figure S12. NIR-II cancer therapy.** CD4 and CD8 in lymph of G1, G2, G3 and G4 (blue channel: 433–468 nm, Ex: 405 nm; red channel: 500–530 nm, Ex:488 nm). Scale bar: 100 µm.


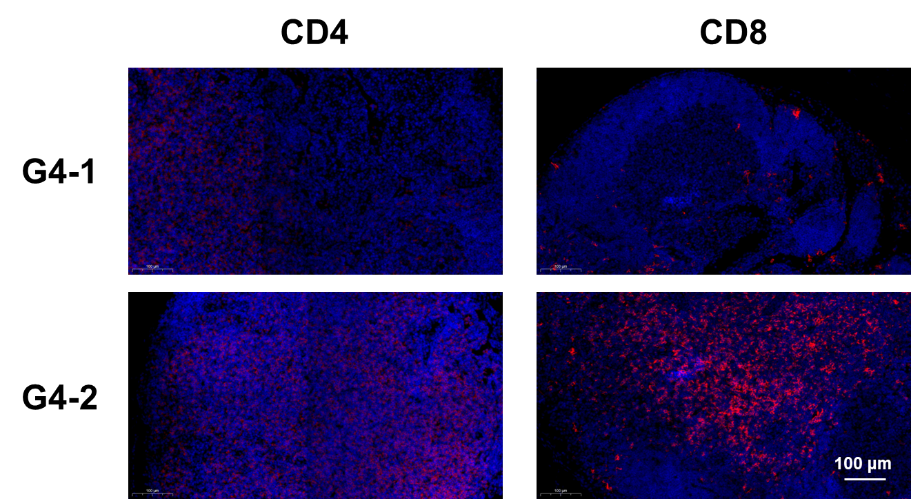


**Figure S13. NIR-II cancer therapy.** CD4 and CD8 in lymph of G4-1 and G4-2 (blue channel: 433–468 nm, Ex: 405 nm; red channel: 500–530 nm, Ex:488 nm). Scale bar: 100 µm.

**References**

1. Roper, D.K., Ahn, W. & Hoepfner, M. Microscale heat transfer transduced by surface plasmon resonant gold nanoparticles. J Phys Chem C **2007**, 111, 3636-3641.
2. L. Zhang, S. C. Wan, J. Y. Zhang, M. J. Zhang, Q. C. Yang, B. X. Zhang, W. Y. Wang, J. W. Sun, R. T. K. Kwok, J. W. Y. Lam, H. X. Deng, Z. J. Sun, B. Z. Tang, *J. Am. Chem. Soc.* **2023**, 145, 17689.
